# Supplementary material for: Exploring the Mystery of the Sweetness of Baijiu by Sensory Evaluation, Compositional Analysis and Multivariate Data Analysis
Source: Foods. 2021 Nov 17;10(11):2843. doi: 10.3390/foods10112843 (PMC8622430; doi:10.3390/foods10112843)
Supplement: Supplementary file 1 [file foods-10-02843-s001.zip › foods-1464642-SI.pdf]

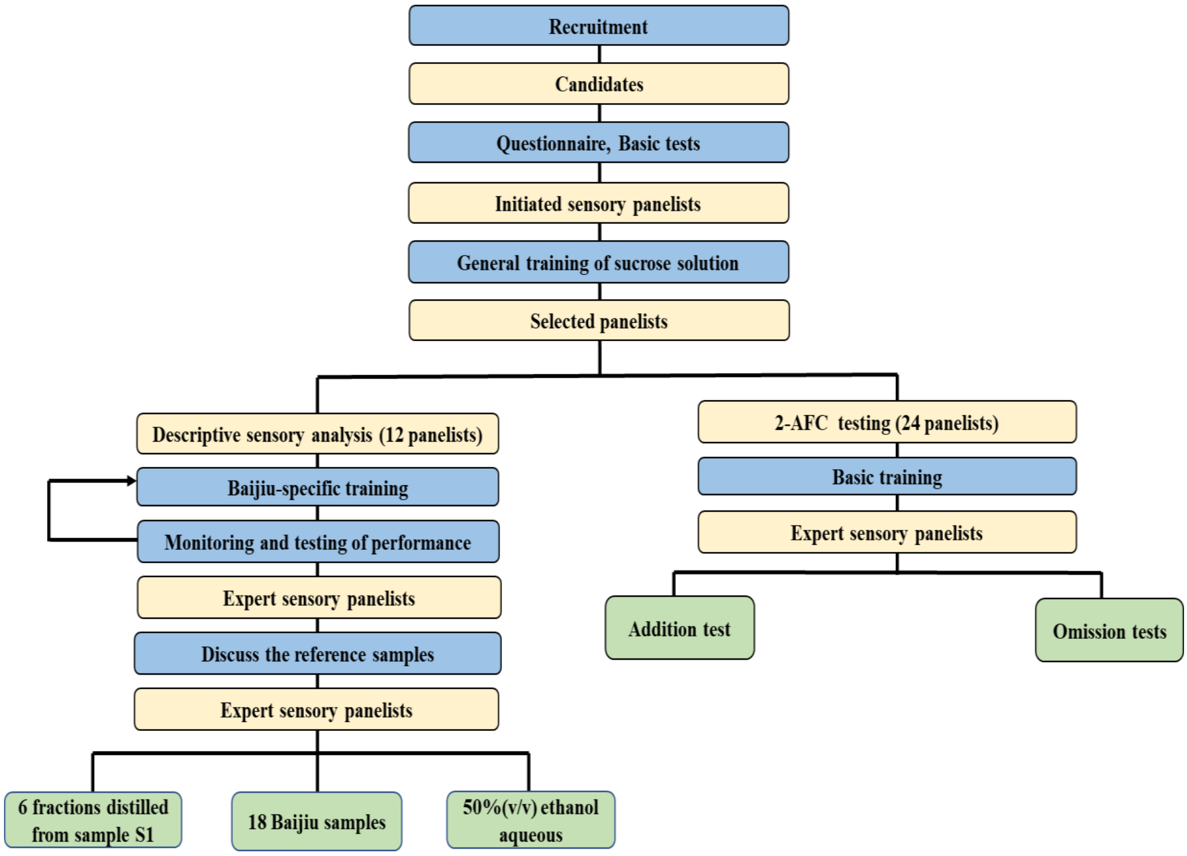

Figure S1. The entire process of sensory evaluation.

Table S1. Details relating to the 18 Baijiu test samples.

Sample	Flavor type	Original ethanol content (%, v/v)	Dilution factor	pH	Origin
S1	Strong flavor	68.8 ± 0.1	1.4	3.60 ± 0.02	Jiangsu, China
S2	Strong flavor	50.4 ± 0.1	1.0	3.66 ± 0.01	Jiangsu, China
S3	Strong flavor	50.2 ± 0.2	1.0	3.58 ± 0.02	Jiangsu, China
S4	Strong flavor	50.8 ± 0.1	1.0	3.93 ± 0.02	Jiangsu, China
S5	Strong flavor	67.3 ± 0.2	1.3	3.59 ± 0.00	Sichuan, China
S6	Strong flavor	52.8 ± 0.1	1.1	3.55 ± 0.01	Sichuan, China
S7	Strong flavor	52.0 ± 0.0	1.0	3.63 ± 0.00	Jiangsu, China
S8	Light-flavor	52.2 ± 0.1	1.0	3.60 ± 0.01	Shanxi, China
S9	Light-flavor	50.0 ± 0.0	1.0	3.88 ± 0.01	Chongqing, China
S10	Light-flavor	54.0 ± 0.2	1.1	3.99 ± 0.01	Beijing, China
S11	Light-flavor	53.0 ± 0.0	1.1	3.64 ± 0.01	Shanxi, China
S12	Sauce-flavor	53.0 ± 0.1	1.1	3.53 ± 0.00	Guizhou, China
S13	Miscellaneous flavor	53.0 ± 0.1	1.1	3.70 ± 0.01	Hubei, China
S14	Miscellaneous flavor	53.0 ± 0.0	1.1	3.74 ± 0.02	Hubei, China
S15	Miscellaneous flavor	53.0 ± 0.2	1.1	3.75 ± 0.03	Hubei, China
S16	Feng flavor	55.0 ± 0.1	1.1	4.58 ± 0.02	Shaanxi, China
S17	Te flavor	52.0 ± 0.2	1.0	4.20 ± 0.01	Jiangxi, China
S18	Medicine-flavor	54.0 ± 0.1	1.1	3.58 ± 0.02	Guizhou, China

Table S2. Analysis of variance of sweetness intensity data of the 18 Baijiu samples.

Source	Degrees of freedom	Sum of squares	Mean squares	F	Pr(>F)[a]
Sample	17	4299	252.89	74.029	< $2e^{-16}$ ***
Panelist	11	136	12.40	3.630	$5.56e^{-05}$ ***
Replication	3	8	2.57	0.752	0.521
Sample × Panelist	187	1023	5.47	1.601	$2.05e^{-05}$ ***
Sample × Replication	51	208	4.09	1.196	0.173
Panelist × Replication	33	148	4.49	1,313	0.116
Residuals	561	1916	3.42		

[a]Significance. codes: 0 '***' 0.001 '**' 0.01 '*' 0.05 '.'

Table S3. Semi-quantitative analysis of aroma compounds in the four distilled fractions made from Baijiu sample S1.

No.	CAS	RI(FFAP)	Compound	m/z	Concentration(ug·L^{-1})[a]			
					Fraction A	Fraction B	Fraction C	Fraction D
			**Esters**					
1	141-78-6	891	Ethyl ethanoate	43	20085.97±1056.49[a]	1519.01±60.96[b]	nd[c]	nd[c]
2	105-54-4	1044	Ethyl butanoate	43	7221.81±833.98[a]	3493.25±158.96[b]	nd[c]	nd[c]
3	108-64-5	1070	Ethyl 3-methylbutanoate	88	1096.38±136.94[a]	nd[b]	nd[b]	nd[b]
4	539-82-2	1128	Ethyl pentanoate	57	4040.75±210.67[b]	4216.65±272.50[a]	21.78±1.41[c]	11.44±2.32[c]
5	123-66-0	1227	Ethyl hexanoate	88	54220.95±3298.92[a]	59366.58±2044.24[a]	5635.67±156.91[b]	1750.49±225.35[b]
6	106-27-4	1289	Isoamyl butanoate	43	147.39±5.62[b]	238.23±1.23[a]	nd[c]	nd[c]
7	142-92-7	1292	Hexyl ethanoate	56	90.65±10.07[b]	103.68±5.32[a]	nd[c]	nd[c]
8	626-77-7	1332	Propyl hexanoate	99	1177.52±70.80[b]	1720.45±83.82[a]	nd[c]	nd[c]
9	106-30-9	1326	Ethyl heptanoate	88	6732.34±340.38[b]	9973.8±397.90[a]	729.8±24.28[c]	66.71±8.29[c]
10	687-47-8	1351	Ethyl 2-hydroxypropanoate	45	nd[b]	nd[b]	nd[b]	2536.75±69.62[a]
11	105-79-3	1363	Isobutyl hexanoate	99	803.65±52.36[b]	1718.93±99.65[a]	nd[c]	nd[c]
12	2050-09-1	1371	Isopentyl pentanoate	70	159.17±17.17[b]	391.56±31.88[a]	nd[c]	nd[c]
13	626-82-4	1422	Butyl hexanoate	56	1517.52±104.50[b]	3551.86±130.25[a]	73.75±1.94[c]	nd[c]
14	106-32-1	1438	Ethyl octanoate	88	11035.85±429.93[b]	21243.45±584.92[a]	2688.69±60.06[c]	143.58±12.34[c]
15	10032-13-0	1468	Hexyl 3-methylbutanoate	85	nd[b]	13.26±0.54[a]	nd[b]	nd[b]
16	2198-61-0	1468	Isoamyl hexanoate	85	23.18±1.20[b]	46.36±1.24[a]	2.87±0.51[c]	nd[c]
17	540-07-8	1514	Amyl hexanoate	70	236.49±16.95[b]	722.47±18.33[a]	nd[c]	nd[c]
18	624-13-5	1521	Propyl caprylate	43	16.54±0.64[a]	nd[b]	nd[b]	nd[b]
19	123-29-5	1537	Ethyl nominates	88	390.62±27.76[b]	1329.18±28.79[a]	nd[c]	nd[c]
20	6946-90-3	1543	Ethyl dl-2-hydroxycaproate	69	nd[b]	103.54±15.48[b]	1725.28±330.35[a]	83.97±5.75[b]
21	5461-06-3	1553	Isobutyl octanoate	57	28.89±4.14[a]	nd[b]	nd[b]	nd[b]
22	6378-65-0	1615	Hexyl hexanoate	43	930.53±63.83[a]	35.35±0.76[c]	508.96±27.41[b]	nd[c]
23	110-38-3	1641	Ethyl decanoate	88	777.74±34.76[b]	2361.44±314.30[a]	nd[c]	nd[c]
24	2035-99-6	1659	Isoamyl octanoate	70	153.1±4.69[b]	639.85±79.74[a]	nd[c]	nd[c]
25	76649-16-6	1668	Ethyl trans-4-decenoate	88	nd[b]	45.18±9.02[a]	nd[b]	nd[b]
26	123-25-1	1680	Ethyl succinate	101	13.04±0.84[b]	12.88±0.36[b]	380.58±8.14[a]	104.63±3.10[b]
27	93-89-0	1685	Ethyl benzene carboxylate	105	36.41±0.24[c]	267.13±20.68[b]	415.55±67.92[a]	nd[c]
28	627-90-7	1739	Ethyl undecanoate	88	19.2±0.27[b]	106.39±14.75[a]	nd[c]	nd[c]
29	101-97-3	1798	Ethyl 2-phenylethanoate	57	199.88±16.32[b]	1180.56±38.49[b]	5251.01±995.04[a]	176.71±5.07[b]
30	1117-55-1	1809	Hexyl octanoate	43	104.88±4.43[b]	321.24±10.47[a]	nd[c]	nd[c]
31	4887-30-3	1810	Octyl hexanoate	117	65.68±2.66[a]	nd[b]	nd[b]	nd[b]
32	106-33-2	1845	Ethyl do decanoate	88	418.78±8.84[b]	758.27±21.18[a]	nd[c]	nd[c]
33	39252-02-3	1871	Furfuryl hexanoate	81	nd[c]	128.05±4.11[b]	432.19±79.68[a]	nd[c]
34	2021-28-5	1902	Ethyl 3-phenylpropanoate	104	125.93±17.34[b]	689.13±23.26[b]	2796.58±497.93[a]	133.56±6.46[b]
35	28267-29-0	1942	Ethyl redecorates	88	10.79±0.69[b]	29.14±0.75[a]	nd[c]	nd[c]
36	103-52-6	1978	2-Phenylethyl butanoate	104	nd[b]	41.95±2.64[b]	312.93±54.08[a]	nd[b]
37	110-27-0	2031	Isopropyl Myristate	43	8.18±1.25[a]	nd[b]	nd[b]	nd[b]
38	124-06-1	2047	Ethyl tetra decanoate	88	498.01±74.61[b]	989.71±114.12[a]	nd[c]	nd[c]
39	2050-23-9	2112	Diethyl suberate	143	nd[c]	nd[c]	8.04±1.07[a]	2.89±0.37[b]
40	41114-00-5	2150	Ethyl pentadecanoate	88	36.42±0.89[b]	68.39±4.32[a]	44.8±4.27[b]	6.98±0.31[c]
41	6290-37-5	2184	2-Phenethyl hexanoate	104	72.46±12.72[c]	317.02±32.90[b]	1561.53±204.19[a]	62.68±1.31[c]
42	628-97-7	2256	Ethyl hex decanoate	88	985.57±21.03[b]	1442.01±151.68[a]	939.96±175.16[b]	301.91±20.40[c]
43	54546-22-4	2285	Ethyl 9-hexadecenoate	55	11.26±0.51[b]	23.18±1.71[a]	28.5±1.61[a]	nd[c]
44	111-62-6	2480	Ethyl Oleate	43	12.14±0.44[a]	14.78±0.41[a]	27.9±2.37[a]	6.58±0.56[a]
45	544-35-4	2530	Ethyl linoleate	67	39.72±1.94[a]	45.48±1.65[a]	53.98±7.43[a]	22.35±2.54[a]
46	84-69-5	2567	Di isobutyl phthalate	149	89.5±1.98[c]	91.67±3.08[c]	413.53±5.75[a]	220.78±23.80[b]
47	84-74-2	2716	Dibutyl phthalate	76	1.79±0.44[b]	3.01±0.15[b]	7.36±0.21[a]	7.54±0.64[a]
			**Alcohols**					
48	71-36-3	1161	1-Butanol	56	89.08±0.00[c]	424.42±13.46[b]	694.58±131.18[a]	nd[c]
49	123-51-3	1213	Isopentyl alcohol	55	nd[b]	1588.65±99.95[a]	2026.48±322.82[a]	74.92±9.64[b]
50	71-41-0	1253	1-Pentanol	42	nd[b]	nd[b]	207.64±40.28[a]	nd[b]
51	543-49-7	1322	2-Heptanol	45	75.05±3.33[c]	582.66±36.68[b]	904.72±164.29[a]	nd[c]
52	111-27-3	1355	1-Hexanol	56	412.19±14.98[bc]	1205.97±40.30[b]	3497.12±641.37[a]	nd[c]
53	104-76-7	1493	2-Ethylhexanol	57	nd[b]	27.42±2.54[a]	nd[b]	nd[b]
54	111-87-5	1557	1-Octanol	56	nd[c]	248.53±6.50[b]	887.95±18.30[a]	nd[c]
55	60-12-8	1927	Phenylethyl Alcohol	91	13.09±2.60[c]	nd[c]	164.34±29.05[b]	455.18±17.12[a]
56	112-53-8	1962	Dodecanal	55	54.22±7.06[a]	21.01±3.53[b]	nd[c]	nd[c]
			**Acids**					
57	64-19-7	1451	Acetic acid	43	102.75±3.01[b]	nd[c]	nd[c]	1082.69±24.16[a]
58	20286-44-6	1503	1-Methylheptyl butyrate	71	nd[b]	97.42±3.50[a]	nd[b]	nd[b]

59	79-09-4	1540	Propanoic acid	74	nd^{b}	nd^{b}	nd^{b}	44.8±0.62^{a}
60	107-92-6	1629	Butanoic acid	58	27.51±0.7^{c}	nd^{c}	883.6±61.16^{b}	1599.08±24.63^{a}
61	503-74-2	1669	Isovaleric acid	60	nd^{c}	nd^{c}	508.52±93.33^{a}	339.69±5.08^{b}
62	109-52-4	1738	Valeric acid	60	nd^{b}	nd^{b}	1312.61±243.72^{a}	1308.46±18.25^{a}
63	646-07-1	1804	4-Methylpentanoic acid	57	nd^{b}	nd^{b}	nd^{b}	50.72±0.40^{a}
64	142-62-1	1857	Hexanoic acid	60	1105.99±201.61^{c}	1355.58±85.97^{c}	14230.18±2014.07^{a}	10109.27±328.41^{b}
65	149-57-5	1945	Ethylhexoic acid	73	14±0.72^{a}	nd^{b}	nd^{b}	nd^{b}
66	111-14-8	1953	Heptanoic acid	60	48.13±1.67^{c}	114.07±4.45^{c}	3000.8±434.97^{a}	1597.18±42.63^{b}
67	124-07-2	2066	Octanoic Acid	60	181.89±7.79^{c}	428.36±15.14^{c}	6222.28±749.81^{a}	2671.36±166.61^{b}
68	112-05-0	2169	Nonanoic acid	60	nd^{c}	nd^{c}	590.86±59.33^{a}	157.87±27.45^{b}
69	334-48-5	2277	n-Decanoic acid	60	7.46±0.30^{c}	20.12±0.70^{c}	759.05±22.39^{a}	287.65±54.26^{b}
70	112-37-8	2382	Undecanoic acid	60	nd^{c}	nd^{c}	10.48±0.54^{a}	4.08±0.48^{b}
71	65-85-0	2457	Benzene carboxylic acid	105	nd^{b}	2.06±0.85^{b}	10.71±0.35^{a}	10.6±1.56^{a}
72	143-07-7	2488	Dodecanoic acid	73	nd^{c}	nd^{c}	87.77±1.83^{a}	36.08±5.51^{b}
			**Aldehydes and Ketones**					
73	124-13-0	1329	Octanal	43	nd^{b}	22.12±2.45^{a}	nd^{b}	nd^{b}
74	124-19-6	1393	Nonanal	57	50.16±4.40^{b}	145.64±5.92^{a}	nd^{b}	9.34±0.19^{b}
75	112-31-2	1512	Decanal	43	nd^{b}	16.45±0.96^{a}	nd^{b}	nd^{b}
76	100-52-7	1541	Benzoic aldehyde	77	nd^{c}	65.09±2.06^{b}	134.63±22.82^{a}	nd^{c}
77	18829-56-6	1552	(2E)-2-Nonenal	43	nd^{b}	28.34±2.94^{a}	nd^{b}	nd^{b}
78	821-55-6	1400	2-Nonanone	43	54.51±6.46^{b}	255.64±7.87^{a}	nd^{b}	nd^{b}
79	112-12-9	1605	2-Undecanone	58	18.18±0.61^{b}	117.25±20.17^{a}	nd^{b}	nd^{b}
80	2345-28-0	2022	2-Pentadecanone	58	13.39±1.66^{b}	30.41±3.25^{a}	nd^{c}	nd^{c}
			**Furans**					
81	3777-69-3	1216	2-Amylfuran	81	24.87±3.17^{b}	nd^{c}	nd^{c}	91.43±2.07^{a}
82	98-01-1	1478	Furfural	96	30.55±2.07^{b}	nd^{b}	694.32±110.50^{a}	45.12±3.64^{b}
83	98-00-0	1666	2-Furanmethanol	98	nd^{b}	nd^{b}	nd^{b}	10.82±0.34^{a}
			**Phenols**					
84	108-95-2	2017	Phenol	94	nd^{b}	nd^{b}	nd^{b}	79.64±1.73^{a}
85	106-44-5	2096	4-Methylphenol	107	11.34±2.24^{c}	nd^{c}	154.99±27.38^{b}	215.51±6.81^{a}
86	123-07-9	2188	4-Ethylphenol	107	nd^{b}	nd^{b}	163.92±28.48^{a}	149.06±6.77^{a}
			**Terpenes**					
87	3796-70-1	1873	Geranyl acetone	43	nd^{b}	nd^{b}	85.39±14.23^{a}	nd^{b}
88	77-53-2	2143	Cedrol	95	nd^{b}	nd^{b}	4.64±0.90^{a}	3.41±0.17^{a}

Values of the concentration are the mean ± SD.

[a]The different letters indicate significant differences at $p < 0.05$.

Table S4. Semi-quantitative analysis of 43 potential sweet compounds in 18 Baijiu samples with different sweetness intensities.

NO.	RI	CAS	Compound	m/z	Concentration(ug·L^{-1})																	
					S1	S2	S3	S4	S5	S6	S7	S8	S9	S10	S11	S12	S13	S14	S15	S16	S17	S18
1	891	141-78-6	Ethyl ethanoate[a]	/	1205857.62±27892.81	829359.82±40308.58	969337.05±121040.52	947989.58±66332.47	1113194.03±42992.46	772407.14±83047.14	265665.57±12708.08	1263372.6±133984.56	505467.9±21208.82	872904.4±61388.62	1225127.58±36946.83	1299174.54±110150.01	775979.1±65142.53	540356.33±35544.47	782134.11±62283.81	1072490.17±67698.60	904043.11±58593.86	1505274.99±219095.69
2	1044	105-54-4	Ethyl butanoate[a]	/	287517.99±7861.45	90803.01±9115.10	199196.41±51135.19	232784.54±41050.71	215342.04±20094.10	172021.19±23181.83	110648.17±7721.29	5334.58±630.06	5407.79±1280.04	3168.38±627.390	6563.17±835.42	91055.58±7723.21	80102.17±24519.71	57418.83±11972.84	84085.09±1573.70	55655.3±7895.56	49141.66±7880.31	310291.93±46098.44
3	1227	123-66-0	Ethyl hexanoate[a]	/	4040807.69±71354.79	1614955.08±176053.02	2320462.26±355850.28	2369996.34±486257.82	2825736.19±89503.30	1617516.59±149462.08	452948.59±86368.44	677236.82±59088.80	419142.44±15911.2	531501.14±25848.39	806470.83±51389.14	452861.58±45246.11	592658.11±132051.29	983420.44±213812.39	1047796.87±263527.41	1167220.05±54529.26	837095.97±87450.55	1146475.69±217832.11
4	1059	108-64-5	Ethyl 3-methyl butanoate[b]	88	103973.73±42384.21	38994.7±8138.37	55235.66±3577.19	90095.27±8507.42	50750.62±1508.18	41648.03±14782.18	16307.04±3654.62	10943.04±5204.76	1002.25±55.02	1709.88±979.45	7213.06±328.64	35952.64±6450.95	44267.97±1932.8	25607.83±1430.55	29691.96±7514.4	6259.95±98.22	31536.73±572.00	44046.29±3346.35
5	1136	539-82-2	Ethyl pentanoate[b]	88	273635.83±60085.17	107840.5±45966.07	146842.4±12528.79	157639.03±16299.87	244202.36±1284.66	128939.85±84170.05	59820.6±9997.16	18211.93±6326.23	3145.97±120.32	13294.96±6906.69	10206.6±4490.12	32402.71±2040.14	59117.43±1578.66	89963.39±11657.99	66227.95±8419.60	83225.73±1338.77	42281.48±4150.48	58267.49±1053.11
6	1391	821-55-6	2-Nonanone[b]	43	10721.14±368.51	21781.72±1189.54	14724.12±590.99	22919.74±10948.54	48843.25±4560.36	10086.81±1884.39	36678.23±4178.92	9199.74±295.24	727.28±16.31	9835.53±224.91	3658.76±777.68	13321.32±1444.58	51023.04±1004.2	50864.83±6978.71	48745.69±3613.44	24549.17±370.18	23049.98±434.24	9100.99±1551.05
7	1292	106-27-4	Isoamyl butanoate[b]	71	21108.74±3061.54	17452.36±2180.6	40288.98±289.16	40353.07±3764.90	29102.57±191.57	13144.22±6572.11	nd	2710.77±155.70	399.14±8.80	3322.66±181.40	1261.97±548.91	3050.28±152.14	13706.19±6853.1	10171.58±5085.90	11442.17±2794.67	24238.69±2023.98	19367.3±7196.63	nd
8	1296	142-92-7	Hexyl ethanoate[b]	61	9454.84±1271.38	5606.93±1272.28	10101.26±5050.63	12889.15±1869.29	3481.34±137.36	3988.07±616.35	747.94±32.91	1317.95±798.69	1649.84±77.28	3457.71±1308.23	1005.73±403.86	1760.31±401.81	2132.45±428.74	2933.03±539.18	1851.19±228.49	19167.15±1576.86	16076.8±559.77	8100.1±2499.74
9	1304	124-13-0	Octanal[b]	43	2110.62±1614.69	3129.56±420.50	6198.53±32.80	9045.72±663.27	13122.19±2891.63	1965.85±908.23	2283.38±149.10	1249.69±684.78	412.5±194.11	1369.06±79.61	1521.51±342.88	1392.7±209.31	4093.11±1432.78	2574.98±372.18	2295.2±833.03	6862.13±249.25	5273.05±543.55	1669±237.27
10	1328	626-77-7	Propyl hexanoate[b]	99	25573.41±1349.54	29200.19±6398.38	46041.1±9566.31	39038.49±1545.32	17771.74±4570.6	10775.54±2028.41	7397.92±1088.00	2443.69±112.44	289.59±143.20	1996.59±780.68	736.74±12.36	10063.41±2497.15	26510.96±96.19	25263.26±3396.74	23320.29±3656.49	40524.16±4158.51	28710.26±1907.60	81365.92±1828.80
11	1343	106-30-9	Ethyl heptanoate[b]	60	93496.91±1704.00	97719.99±22314.40	135582.61±16375.89	105237.82±36784.79	62212.53±1100.12	44657.91±6882.55	44659.58±3500.02	13244.44±5322.67	1373.77±191.7	8712.29±2439.55	8543.51±218.04	16070.09±459.33	43896.54±1800.14	42750.47±3629.95	37335.55±2720.02	89767.18±3778.92	33926.6±2383.89	47560.6±12298.81
12	1357	105-79-3	Isobutyl hexanoate[b]	99	26063.24±3958.60	26075.76±3807.35	43837.74±8206.31	34446.64±3184.22	22843.49±4217.53	12608.68±5215.30	6895.81±675.81	2293.93±106.40	270.8±3.54	1128.63±438.38	750.21±30	3478.87±630.06	16421.85±1052.74	14540.43±2466.21	14083.45±909.65	32851.27±1693.59	22621.81±1120.85	21413.26±3061.97
13	1363	2050-09-1	Isopentyl pentanoate[b]	70	12799.03±463.10	10034.81±893.32	25692.8±6198.59	17941.24±448.05	9144.04±103.56	5055.7±1544.64	1237.16±86.17	652.3±36.89	nd	521.06±18.68	245.63±79.13	3835.23±424.75	6214.09±1100.33	4650.21±204.42	5293.18±536.39	13800.14±288.78	13826.6±860.90	17915.02±2260.69
14	1395	124-19-6	Nonanal[b]	57	9411.15±596.47	8071.92±610.85	13810.47±2625.39	10747.01±960.53	8679.72±831.12	4441.17±167.94	6614.7±571.33	6746.9±2026.72	2699.68±157.09	7824.02±571.60	8829.22±213.78	8485.25±401.65	8528.66±788.18	7011.74±379.69	8800.2±405.68	25211.96±3219.7	14082±711.70	8513.48±175.66
15	1414	626-82-4	Butyl hexanoate[b]	56	27877.91±11975.98	84920.94±8209.42	144926.07±28696.18	92627.74±2941.25	56009.93±5129.54	37764.45±1295.00	17112.54±2344.49	8223.5±240.31	232.36±14.93	3439.88±139.35	1287.83±40.95	15379.81±73.80	21836.22±1422.27	28845.48±4823.25	22142.39±2639.79	114250.3±4276.13	48996.55±1936.32	75786.78±1689.36
16	1435	106-32-1	Ethyl octanoate[b]	88	268979±45504.87	124083.66±37604.64	188047.88±60977.22	173904.02±14149.54	109299.5±23554.79	105666.06±15398.74	138465.42±27657.91	105551.99±54476.88	57547.26±17901.17	80818.73±29967.69	61551.94±1093.51	86453.98±37552.99	105963.95±13563.72	135296.22±15157.88	128515.32±11111.00	183714.64±44887.10	142886.34±24317.63	117288.41±34292.16
17	1441	10032-13-0	Hexyl 3-methyl butanoate[b]	85	959.98±39.56	727.22±26.46	nd	1535.43±166.34	545.13±122.87	2541.36±501.06	73.62±0.13	19.62±1.21	nd	nd	nd	175.72±16.94	321.4±14.23	1336.09±271.99	246.66±29.46	5109.74±284.92	656.05±27.52	2469.76±172.16

18	1460	2198-61-0	Isoamyl hexanoate[b]	43	31106.15±2554.7	82179.02±1126.16	6251.41±1701.99	70864.53±1672.50	54877.43±1326.2	44798.59±5765.6	28949.9±1753.98	11776.28±4205.63	779.7±165.89	5175.39±130.75	4379.97±107.91	12624.97±3033.61	47049.45±2726.85	43474.68±3873.51	45063.52±3041.98	38550.53±2889.19	29938.07±2105.22	53075.23±5707.4
19	1480	104-76-7	2-Ethylhexanol[b]	57	4430.66±331.13	1972.54±164.69	1423.71±3.16	11108.95±621.02	1854.18±227.06	1706.08±263.42	879.59±79.94	1326.86±450.73	1054±383.08	1343.89±106.86	4662.98±433.09	2279.96±126.24	1273.8±67.12	1417.18±60.55	1345.1±94.13	1589.13±69.83	2629.13±99.1	1021.03±20.39
20	1489	20286-44-6	1-Methyl heptyl butyrate[b]	71	nd	5330.82±307.54	nd	1038.31±162.92	7470.31±100.27	206.76±10.49	2485.12±325.59	225.31±10.36	nd	nd	nd	nd	448.7±22.35	2403.63±58.07	nd	898.7±15.38	701.28±350.64	560.97±36.65
21	1497	112-31-2	Decanal[b]	43	2219.22±16.73	2019.62±25.96	2295.31±114.66	4036.79±422.99	1837.92±23.03	1261.26±212.58	1143.03±11.05	1925.35±694.88	493.92±37.43	1322.98±183.83	1806.17±162.77	1535.47±168.91	3118.28±94.31	2181.73±241.51	2618.34±175.38	4465.44±148.58	4793.65±238.29	2644.25±118.94
22	1506	540-07-8	Amyl hexanoate[b]	43	29379.52±4454.55	30894.82±15447.41	38975.33±8517.28	30213.26±3221.70	18706.64±1752.24	19456.82±6584.83	5760.94±317.06	1622.42±573.52	nd	nd	398.12±19.06	4032.87±673.5	7349.13±234.86	14234.05±2502.09	5233.09±119.80	35592.57±1044.33	16473.82±7585.98	30253.87±7027.45
23	1505	18829-56-6	(2E)-2-Nonenal[b]	70	nd	62.84±4.89	1399.8±131.69	nd	65.17±4.87	nd	894.84±44.42	25.12±13.15	144.64±5.64	nd	nd	nd	29.13±1.56	nd	nd	1360.77±127.06	nd	nd
24	1512	624-13-5	Propyl caprylate[b]	61	9107.11±200.34	8628.79±216.54	11755.23±3503.00	10068.57±692.41	3368.35±1.17	2774.82±839.31	987.59±10.58	671.13±29.39	194.87±50.91	608.92±142.16	434.72±81.12	1989.65±167.14	3536.2±71.98	2814.74±109.18	2944.64±229.44	10211.48±538.89	5288.2±39.84	29033.95±5520.56
25	1530	123-29-5	Ethyl nonanoate[b]	88	44898.25±2937.97	40755.21±4822.91	41921.32±1082.55	40491.35±4507.37	39525.05±3962.25	32130.98±3730.99	28438.54±1505.88	34457.49±8609.98	3335.43±729.32	37023.13±7819.84	35795.16±7207.00	25596.45±3781.12	28278.95±1250.17	33062.68±8358.11	25890.6±1324.52	57628.11±1396.28	60538.02±580.93	32400.86±5391.43
26	1593	112-12-9	2-Undecanone[b]	58	5458.5±534.12	11510.17±21.11	9013.8±236.42	11684.1±1964.26	46066.25±3960.56	4137.1±523.90	3671.79±341.83	5647.35±587.85	334.92±112.80	7146.59±107.01	2623.1±551.26	16159.56±765.76	19791.4±1055.33	18425.1±2948.90	18300.87±915.92	14447.66±333.47	18960.04±1083.19	7726.21±787.46
27	1602	6378-65-0	Hexyl hexanoate[b]	117	98242.5±4629.92	87213.89±1010.26	88789.94±2955.82	65269.97±9759.67	55965.94±1076.59	79300.22±1009.64	25844.57±1003.12	6207.4±136.41	252.47±189.53	2600.23±962.75	2195.8±140.58	9280.58±229.15	24362.44±1287.82	33174.71±6720.31	21865.31±871.68	67423.92±9465.96	33753.54±1816.2	53829.02±8118.34
28	1606	5461-06-3	Isobutyl octanoate[b]	145	nd	nd	nd	nd	nd	nd	nd	nd	nd	nd	nd	nd	nd	nd	276.86±7.86	nd	nd	nd
29	1630	110-38-3	Ethyl decanoate[b]	101	31553.29±2952.84	30820.14±298.64	27586.79±3479.28	23069.43±709.00	35072.16±5388.84	23338.06±3208.11	17124.72±1050.66	41687.83±1181.61	18998.14±6499.18	42211.88±1033.87	37807.34±991.6	20417.31±2720.74	21402.74±1073.55	24725.44±7185.46	21923.79±818.97	25511.54±2280.42	34569.15±5560	52569.39±931.17
30	1650	2035-99-6	Isoamyl octanoate[b]	70	27447.49±1190.85	24136.82±4974.39	30951.86±8628.62	21201.64±4096.69	10133.13±1965.12	6086.88±766.65	3894.63±166.11	3000.05±316.81	578.64±19.89	2775.63±144.71	3081.09±538.46	1287.45±74.73	2629.3±326.12	3176.28±453.15	2800.48±172.08	7849.02±531.13	4501.08±396.83	7891.6±519.20
31	1657	76649-16-6	Ethyl trans-4-decenoate[b]	88	nd	nd	nd	nd	nd	nd	nd	nd	nd	nd	nd	7233.34±890.02	2313.83±70.38	nd	nd	3540.21±53.63	4864.03±17.48	3022.26±341.81
32	1733	627-90-7	Ethyl undecanoate[b]	88	5488.92±153.88	2990.32±517.72	4279.98±156.94	4013.55±830.92	4037.07±942.98	2074±125.21	1362.91±140.88	1351.57±25.91	56.77±30.51	2122.52±550.15	1255.46±182.69	1312.56±169.06	1425.79±212.42	2117.01±341.52	1627.56±126.27	3221.28±135.16	7284.43±268.2	3876.75±128.49
33	2012	2345-28-0	2-Pentadecanone[b]	58	nd	nd	nd	nd	nd	nd	nd	nd	nd	296.15±11.51	97.36±4.68	1393.37±37.16	nd	nd	nd	nd	7091.87±73.70	nd
34	1799	1117-55-1	Hexyl octanoate[b]	43	9825.3±709.77	11523.13±1997.71	19117.83±8456.23	15068.07±277.64	7327.84±473.25	5332.16±109.42	1499.18±451.51	190.62±34.47	nd	130.81±18.37	77.39±10.84	nd	nd	629.12±28.41	nd	nd	3106.27±237.03	nd
35	1806	4887-30-3	Octyl hexanoate[b]	117	8885.9±177.96	nd	nd	nd	nd	nd	nd	nd	nd	nd	nd	222.8±30.98	395.04±11.55	267.95±13.98	317.12±15.56	nd	nd	nd
36	1840	106-33-2	Ethyl dodecanoate[b]	88	30594.67±3074.62	30234.16±2212.27	31434.45±8566.99	34210.03±5666.24	23813.64±685.37	12706.09±1579.34	14390.52±992.15	29057.19±5643.28	11311.1±565.55	35918.66±1703.49	30806.85±5481.19	24161.62±4255.55	14545.5±898.84	24980.02±7076.67	16848.15±352.92	18649.31±1294.91	59327.3±5153.26	40644.78±4131.59
37	1935	28267-29-0	Ethyl tridecanoate[b]	88	650.66±14.23	874.67±43.33	893.68±36.97	704.94±12.82	1184.61±59.3	nd	nd	nd	nd	340.65±17.33	34.32±1.16	417.44±60.05	nd	523.33±26.67	nd	nd	2749.73±133.18	nd

38	1937	149-57-5	Ethylhexoic acid[b]	73	580.97±29.48	446.27±22.28	362.47±18.23	453.34±11.01	196.37±9.18	230.42±46.53	205.2±28.65	413.99±10.89	208.75±10.17	nd	319.38±14.27	109.88±27.74	154.38±77.19	140.2±10.89	196.21±8.42	187.58±12.21	358.8±18.70	442.05±12.30
39	1952	112-53-8	Dodecanol[b]	55	525.93±61.06	1593.85±820.01	362.47±18.23	599.67±51.04	1798.47±86.45	864.01±14.50	2111.09±938.5	1138.84±374.24	710.28±54.38	924.67±25.87	648.16±14.90	766.71±25.96	1194.73±232.22	1617.62±66.61	1353.77±315.92	2315.5±462.45	1197.64±74.55	1515.44±38.99
40	2023	110-27-0	Isopropyl Myristate[b]	43	nd	203.83±60.12	77.14±3.57	77.23±11.43	352.5±16.99	108.21±54.11	164.5±10.6	nd	68.65±3.32	259.45±12.73	333.13±16.57	133.09±61.61	107.05±45.74	149.75±7.36	104.06±4.12	205.54±3.60	nd	301.37±58.52
41	2040	124-06-1	Ethyl tetradecanoate[b]	88	20927.64±4327.44	33149.49±731.01	28769.4±7828.59	31405.88±5403.29	30463.61±8647.73	5951.09±401.94	6455.24±668.67	5771.98±667.08	346.9±173.45	11169.8±3116.59	3397.61±403.4	21894.65±4818.94	13361.11±110.49	25654.07±3579.15	16287.07±1559.97	3662.4±238.19	114109.39±11940.91	21965.01±1005.47
42	2139	41114-00-5	Ethyl pentadecanoate[b]	88	1188.38±148.88	2463.8±123.90	2657.27±132.63	1278.95±7.87	1716.8±545.27	nd	nd	208±17.06	nd	987.92±37.89	nd	4349.6±107.66	nd	1542.8±113.34	nd	nd	7523.35±913.45	2432.54±121.27
43	2242	628-97-7	Ethyl hexadecanoate[b]	88	37103.36±943.25	53478.93±8999.47	43867.86±2156.47	50437.1±5245.11	40107.55±8266.59	15548.46±2003.52	15368.81±394.42	12696.8±1667.36	4422.42±1061.45	32442.45±9763.11	8045.32±108.87	83727.39±1163.53	13348.37±1332.60	39458.25±1640.42	12299.46±1223.19	8825.16±996.92	87625.54±6389.3	41685.52±7162.83

[a]These compounds were quantitated by GC-FID.

[b]These compounds were quantitated by GC × GC−TOFMS/ head-space analysis.

Table S5. Quantitative analysis of the 14 potential compounds which may contributes to sweetness of Baijiu in sample S1 that diluted to 50% ethanol content.

No.	CAS	RI (FFAP)	compound	Liner threshold (ug·L-1)	M/Z	Slope	Intercept	Coefficient of determination	LOQ (ng·L-1)	LOD (ng·L-1)	Accuracy (%)	Concentration (mg·L-1)
1	539-82-2	1136	Ethyl pentanoate[1]	7250.00-232000.00	—	1.3042	0.0016	0.9996	—	—	99.41	59.40±0.00
2	123-66-0	1253	Ethyl hexanoate[1]	39040-9993000	—	1.4763	0.0533	0.9999	—	—	99.55	2172.72±0.19
3	1117-55-1	1798	Hexyl octanoate[2]	110.61-14850.60	84	0.2630	-0.1021	0.9918	—	—	114.38	4.15±0.13
4	108-64-5	1058	Ethyl 3-methylbutanoate[2]	150.94-20400.00	88	0.1035	-0.0169	0.9968	2396.69	719.01	102.15	16.09±0.85
5	106-30-9	1343	Ethyl heptanoate[1]	25900.00-485000.00	—	1.3013	0.0169	1	2863.31	858.99	99.98	40.01±0.00
6	106-27-4	1291	Isoamyl butanoate[2]	10.59-4080.00	71	0.3214	-0.0240	0.9934	—	—	102.75	2.50±0.12
7	6378-65-0	1601	Hexyl hexanoate[2]	2060.25-132000.00	117	0.1488	-0.5738	0.9962	646.34	193.90	100.38	113.58±3.84
8	2035-99-6	1650	Isoamyl octanoate[2]	78.60-10062.10	127	0.5528	-0.1247	0.9916	24320.76	7296.23	117.29	2.01±0.02
9	105-79-3	1357	Isobutyl hexanoate[2]	160.24-10390.20	99	0.3911	-0.1138	0.9942	28071.43	8421.43	92.99	6.13±0.30
10	105-54-4	1028	Ethyl butanoate[1]	6650.00-850750.00	—	1.3013	0.0169	0.9982	4891.57	1467.47	98.63	225.76±0.03
11	2198-61-0	1459	Isoamyl hexanoate[2]	6975.00-223200	70	0.2933	-0.4314	0.9979	—	—	113.28	46.42±2.03
12	106-32-1	1434	Ethyl octanoate[1]	13760.00-440250.00	—	1.4974	-0.0144	0.9998	52848.49	15854.55	100.19	76.89±0.01
13	124-13-0	1300	Octanal[2]	4.54-2317.91	43	0.1516	0.0864	0.9963	—	—	100.06	1.98±0.13
14	540-07-8	1509	Amyl hexanoate[2]	3875.50-31000.00	70	0.1275	-0.0791	0.9903	11.15	3.35	109.89	10.76±0.33

[1] These compounds were quantified by GC-FID.

[2] These compounds were quantified by GC-MS/head-space analysis.
